# Effect of Polyphosphorylation on Behavior of Protein Disordered Regions

**DOI:** 10.3390/ijms22157883

**Published:** 2021-07-23

**Authors:** Pavel I. Semenyuk

**Affiliations:** Belozersky Research Institute of Physico-Chemical Biology, Lomonosov Moscow State University, 119234 Moscow, Russia; psemenyuk@belozersky.msu.ru

**Keywords:** protein polyphosphorylation, inorganic polyphosphate, PASK-motif, post-translational modification, HSP90, endoplasmin

## Abstract

Proteins interact with many charged biological macromolecules (polyelectrolytes), including inorganic polyphosphates. Recently a new protein post-translational modification, polyphosphorylation, or a covalent binding of polyphosphate chain to lysine, was demonstrated in human and yeast. Herein, we performed the first molecular modeling study of a possible effect of polyphosphorylation on behavior of the modified protein using replica exchange molecular dynamics simulations in atomistic force field with explicit water. Human endoplasmin (GRP-94), a member of heat shock protein 90 family, was selected as a model protein. Intrinsically disordered region in N-terminal domain serving as a charged linker between domains and containing a polyacidic serine and lysine-rich motif, was selected as a potent polyphosphorylation site according to literature data. Polyphosphorylation, depending on exact modification site, has been shown to influence on the disordered loop flexibility and induce its further expanding, as well as induce changes in interaction with ordered part of the molecule. As a result, polyphosphorylation in N-terminal domain might affect interaction of HSP90 with client proteins since these chaperones play a key role in protein folding.

## 1. Introduction

Protein interaction with charged polymers (so-called polyelectrolytes), including highly charged proteins and other biological macromolecules [1], is of special relevance for many key processes. One of “classic” polyelectrolytes is inorganic polyphosphates, which were found in many organisms. The exact role of polyphosphates is unclear. Storage of inorganic phosphate, energy resource, chelation of metal ions, role in stress response and other metabolic processes were addressed to polyphosphates as probable functions [2,3,4]. In addition, polyphosphates can exhibit chaperone activity [5] and probably play this role in cells [6].

Surprisingly, polyphosphates can be covalently bound to proteins via lysine residue [7]. Such modification, polyphosphorylation, was demonstrated for around two dozen proteins in yeast and human [7,8,9]. Although information about this modification and preferable modification sites is very poor, it was suggested to occur in polyacidic serine- and lysine-rich regions of the proteins (so-called PASK-motifs). The mechanics of polyphosphorylation seem to differ from two other phosphate-associated modifications, single phosphorylation or pyrophosphorylation, the first of which generally relate to serine, threonine, or tyrosine residues [10] (though phosphorylation of basic residues, arginine and lysine, is also possible [11]), and the second one requires single-phosphorylated serine residue [12]. The known data about polyphosphorylation, which is a non-enzymatic post-translational modification, and some guesses about its probable role are summarized in recent reviews [13,14,15].

**Figure 1:**
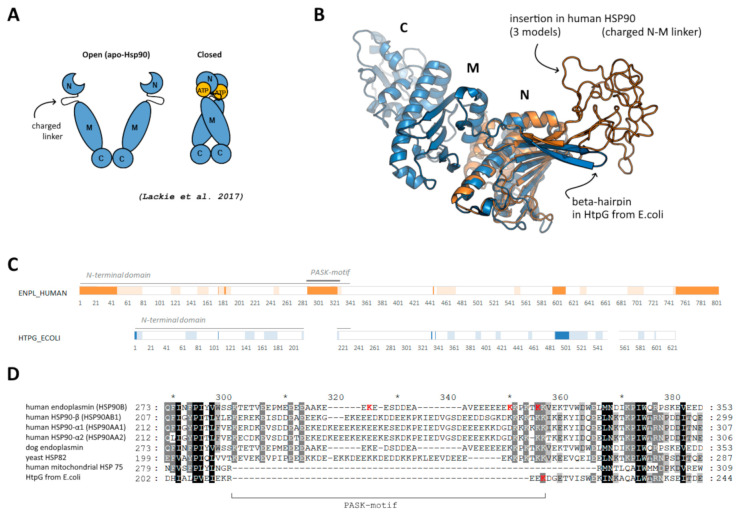
(**A**) Schematic representation of open and closed forms of HSP90, adopted from [18]. (**B**) Location of disordered insertion of PASK-motif in beta-hairpin in N-terminal domain of human endoplasmin (orange) compared to “short” beta-hairpin of bacterial HSP90 (blue); three different initial models are superimposed; middle and C-terminal domains of human endoplasmin are not shown. (**C**) Disordered regions predicted using DISOPRED3 [19] (bright orange/blue) and PONDR [20] (light orange/blue). (**D**) Alignment of the region of the beta-hairpin with the insertion and PASK-motif; reconstructed using Clustal Omega.

Members of Heat shock protein 90 family, some of which are shown to be polyphosphorylated in a disordered PASK-region [7], were selected as a model to study the possible effect of polyphosphorylation. This is a chaperone important for protein folding and maintenance [16], which functions in a dimeric form and undergoes opening/closing during ATP hydrolysis cycle via interaction of N-terminal domains (Figure 1A). All eukaryotic HSP90 members (including human endoplasmin), in contrast to bacterial (such as HtpG from *E. coli*) and mitochondrial homologs, contain prolonged disordered N- and C-ends as well as a prolonged charged linker in the C-terminal region of the N-terminal domain (Figure 1B,C). This inter-domain charged linker (287–327 a.a. in human endoplasmin), which is rich in acidic residues and lysine residues and represents a so-called PASK-motif, plays an important role in structural rearrangement of domains during opening/closing cycle and interaction with client proteins [17]; it was earlier shown to be polyphosphorylated. On the contrary, bacterial HSP90 members have only a short beta-hairpin with few lysine residues (Figure 1D). In the present work, we focused on this region of human endoplasmin and used also HtpG from *E. coli* to compare a possible effect of polyphosphorylation if the protein does not contain PASK-motif, suggesting that the mentioned above lysine residues (probably) might be polyphosphorylated.

In a situation of a lack of information about polyphosphorylation sites in vivo, we suggest modeling studies as a method of choice to predict a possible effect of the modification. Unfortunately, the polyphosphorylated regions are absent in crystal structures since PASK-motif is very acidic and disordered. This is true for eukaryotic HSP90 members, and we reconstructed them using homology modeling. In spite of a high importance of the disordered regions, especially the mentioned insertion which serves as a charged linker between N- and M-domains, for chaperone functioning, we suggested as a main hypothesis, that polyphosphorylation of disordered loop might influence the behavior of this loop or its ordering/further disordering.

## 2. Methods

Molecular dynamics simulations were performed in GROMACS 2018.8 software [21] using CHARMM36m force field [22]. Polyphosphorylated lysine was parameterized using CGenFF service [23]. To avoid artificial crystallization of polyphosphate and counterions observed for sodium ions [24], methylammonium CH_3_NH_3_^+^ was used as a counterion. In all cases, the simulation box contained a molecule of native or polyphosphorylated protein, counterions, and water.

For simulations with bacterial HSP90 from *E. coli*, a structure of chaperone protein HtpG was used (PDB ID 1y4u). Lys216 was manually modified by addition of 10 phosphate groups. All main classic molecular dynamics simulations were of 100 ns with a time step of 2 fs. Periodic boundary conditions and the particle mesh Ewald method for handling long-range electrostatic interaction were used. The simulations were conducted using an NPT ensemble. V-rescale thermostat (300 K) and Parrinello-Rahman barostat were used.

Structure of full-length N-terminal domain of human HSP90 was reconstructed using homology modeling in SWISS-Model [25]. Structures 4nh9 (from human), 1yt2, and 1qye (from dog) were used as templates to obtain three models of disordered loop 287–327, which is absent in PDB structures, as well as the region 163–173, which is also absent in PDB structure of human endoplasmin; the region 65–72 located in N-end of the crystal structure was ignored. These models were energy minimized and then used as initial structures for replica-exchange molecular dynamics simulations. These three structures were randomly distributed among 60 replicas in a temperature range of 300–380 K. Replica exchange attempts were performed every 500 steps; exchange probability was 0.20. The parameters of the simulations were similar parameters to those for classic MD for HSP90 from *E. coli*. Simulation length was 50–65 ns, i.e., total REMD simulation length was more than 3 µs for each system.

## 3. Results

**Figure 2:**
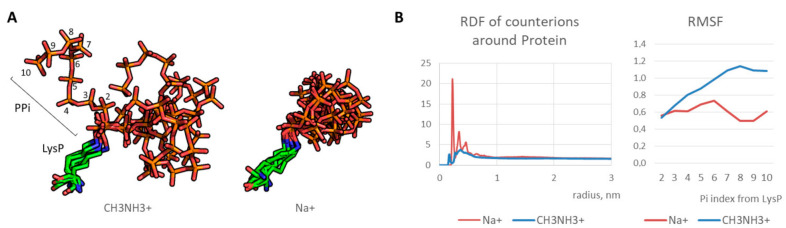
(**A**) Typical states of LysP-PP_i_ chain for 50–100 ns (every 10 ns): stable position in case of Na^+^ and dynamic state in case of CH_3_NH_3_^+^; (**B**) radial distribution function (RDF) graphs for counterions and mobility of PP_i_ residues (in terms of root mean square fluctuation, RMSF).

First, optimal parameters for molecular modeling of the polyphosphorulated proteins were selected. Since polyphosphate chain covalently added due to polyphosphorylation is a highly charged polymer with a high charge density, molecular dynamics simulations require correct modeling of counterions. We tested two types of monovalent cations: sodium cation Na^+^ and methylammonium cation CH_3_NH_3_^+^. An artificial “crystallization” of sodium cations was observed as demonstrated in our previous works on modeling of synthetic polymers with a high charge density [24,26]. On the contrary, methylammonium cation interacted with polyphosphate normally (Figure 2A), indicating a realistic modeling of electrostatic interaction. As a result, polyphosphate chain was flexible in contrast to artificial freezing observed in case of conventional sodium cations. Indeed, radial distribution function for counterions around polyphosphorylated protein molecule is realistic in case of methylammonium cation in contrast to that of sodium cation, and RMSF graph indicates an increase of the phosphate fluctuation with growing phosphate group index (i.e. a distance from the phosphate group and the modified lysine) in contrast to frozen chain in the case of sodium cations (Figure 2B). Therefore, methylammonium cation can be used as a monovalent counterion for realistic modeling of polyanions with high charge density.

**Figure 3:**
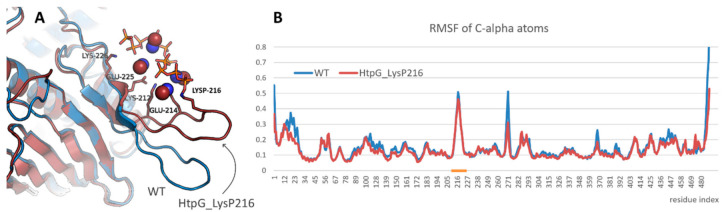
(**A**) Interaction of polyphosphate chain in polyphosphorylated form of HtpG from *E. coli* and position of the modified beta-hairpin compared with that in native form of the protein (blue). Protein, polyphosphate chain, and methylammonium cations that participated in the interaction are shown in cartoon, sticks, and spheres representation, respectively. (**B**) Root mean square fluctuation profiles for native and polyphosphorylated forms of HtpG from *E. coli*. Beta-hairpin region is highlighted with orange.

A study of the effect of polyphosphorylation on protein behavior was started from the modeling of bacterial HSP90, namely, HtpG from *E. coli*. Lys216 residue located in the mentioned above beta-hairpin was polyphosphorylated. It resulted in some rearrangement of the beta-hairpin and formation of new contacts (Figure 3A). Thus, the charged residues located in the beta-hairpin (Lys212, Glu214, Glu225, Lys226 and others) formed ion pairs with polyphosphate chain or with methylammonium cations bound to polyphosphate chain. However, the structure of N-terminal domain of HtpG was not affected. Polyphosphorylation also did not induce any significant changes in the beta-hairpin flexibility (Figure 3B).

**Figure 4:**
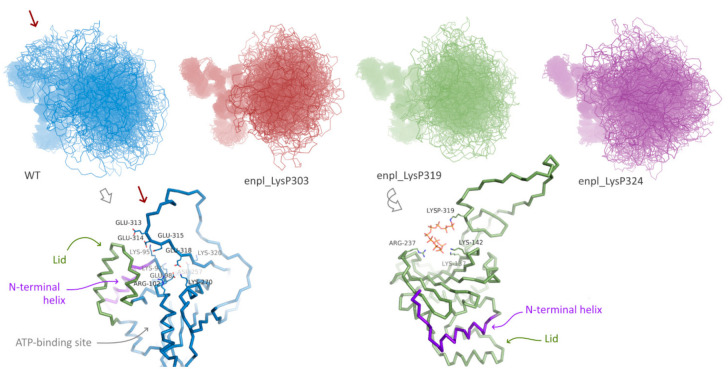
Conformation ensembles obtained using REMD simulations N-terminal domain of native endoplasmin and three polyphosphorylated forms: LysP303, LysP319, and LysP324. Red arrow indicates additional interaction of the disordered charged linker with globular part of N-terminal domain in native endoplasmin; example of such interaction is presented in detail.

Effect of polyphosphorylation on behavior of human HSP90, namely, endoplasmin, was much more pronounced. Three forms polyphosphorylated at Lys residues located in the disordered insertion in the beta-hairpin were studied: polyphosphorylation at Lys303, Lys319 and Lys324 (Figure 1D). For all these forms and the native one, replica exchange molecular dynamics simulations were performed. In all cases, the globular part of N-terminal domain of endoplasmin was stable, whereas the disordered loop of the charged linker was flexible (Figure 4). A significant difference between conformational ensembles of this disordered loop in native and polyphosphorylated forms was observed. In the native form, Glu313, Glu314, Glu315, Glu318 and other acidic residues interact with positively charged residues of the globular part of N-terminal domain, specifically, N-terminal helix (marked with red arrow in Figure 4, see a general and detailed view on bottom-left). The double-helix loop 166–196 (colored in purple in Figure 4), serving as a lid for ATP-binding pocket, is also very close, although direct interaction between the lid and charged linker was rare in the conformational ensemble of the native form of endoplasmin. These two elements (N-terminal helix and the lid 166–196) play a key role in structural rearrangement due to opening/closing cycle of the yeast HSP90 and its regulation by co-chaperone p23 [27]. Surprisingly, further acidification of this part of the charged linker via polyphosphorylation of adjacent lysine residues resulted in a complete inhibition of such interaction instead of expected enhancing. This effect might arise from a possible role of the mentioned lysine residues in the interaction or from significant changes in general behavior of the disordered charged linker caused by polyphosphorylation.

**Figure 5:**
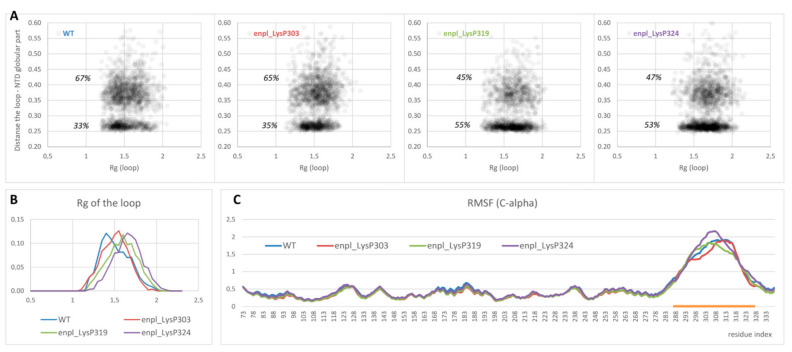
(**A**) Distribution of conformations in the space (R_g_ of the disordered loop; minimum distance between the loop and globular part of the N-terminal domain) obtained from REMD ensemble. (**B**,**C**) R_g_ distribution (**B**) as well as RMSF profiles of C-alpha atoms (**C**) for the same systems. The region of the loop is highlighted with orange.

On the other hand, overall affinity of the disordered loop to globular part of N-terminal domain in two polyphosphorylated forms (enpl_LysP319 and enpl_LysP324) increased compared to the native form. Thus, a percentage of conformations with the distance between the loop and the globular part lower than 0.3 nm (in other words, with the contacts between the loop and the globular part) increased from 33% for the native form to 53–55% for these polyphosphorylated forms (Figure 5A). In these polyphosphorylated forms, enpl_LysP319 and enpl_LysP324, polyphosphate chain directly interacted with a back side of the beta-sheet via Arg237, Lys137 and Lys142 (Figure 4, bottom-right); some interaction with N-terminal strand was also detected. As to the third polyphosphorylated form, enpl_LysP303, polyphosphate chain interacted with a globular part of the N-terminal domain significantly more rarely.

Polyphosphorylation caused some expanding of the disordered loop: the radius of gyration of the loop increased in all polyphosphorylated forms compared to the native from (Figure 5B). It was true for both sets of conformations, interacting with the globular part and non-interacting ones (compare density of points with R_g_ < 1.5 and R_g_ > 1.5 in Figure 5A). Effect of polyphosphorylation at Lys319 and 324 was more pronounced compared to modification at Lys303.

Finally, flexibility of the disordered charged linker was estimated from root mean square fluctuation (RMSF) profiles for C-alpha atoms. The values for globular part of N-terminal domain were not affected by polyphosphorylation, and the structure of this part of the protein was not changed. The disordered charged linker was much more flexible in all forms of endoplasmin. Some changes in flexibility were observed for polyphosphorylated forms compared to the native one (Figure 5C), although the difference is not big and does not allow to judge about a general effect of polyphosphorylation on the loop flexibility. However, our data unambiguously indicates the difference in the charged linker behavior, including different occupied conformations and changes in interaction of the charged linker with another part of the protein, especially the N-terminal helix and strand.

## 4. Discussion

Summarizing, we performed the first modeling study of molecular details of a possible role of polyphosphorylation on the protein behavior. Since the intrinsically disordered acidic region, so-called PASK-motif, might be the most probable polyphosphorylation site, we focused our attention on the polyphosphorylation effect on the conformation ensemble of such region in human HSP90 chaperone, namely, endoplasmin, which was shown to undergo this post-translational modification. The disordered PASK-motif is a main part of charged linker between N-terminal and middle domains of HSP90. Using replica exchange molecular dynamics simulations, we clearly demonstrated that polyphosphorylation might change the conformation ensemble of the modified disordered loop and its interaction with other parts of the protein.

HSP90 chaperones are important for protein folding and maintenance in Eukaryotes, although the mechanism of their functioning is poorly understood. The charged linker comprising a disordered PASK-motif connects N-terminal domain, which contains ATP-binding site, and middle domain. Since this prolonged disordered linker plays a key role in HSP90 machinery during closing/opening cycle [17,27], polyphosphorylation of the charged linker might be important for the HSP90 functioning. Indeed, polyphosphorylation has been demonstrated to reduce interaction of the charged linker with key structural elements of globular part of the N-terminal domains, including N-terminal helix, and might indirectly influence the behavior of the ATP-binding pocket lid. On the other side, for two of three tested polyphosphorylated forms, a weak direct interaction of polyphosphate chain with N-terminal strand was detected, that may modulate structural rearrangement or intra-subunit interaction in a HSP90 dimer, which seems to involve swapping of two N-terminal strands [27].

In addition, polyphosphorylation of the charged linker might influence the interaction of HSP90 with other proteins, including client proteins. Indeed, the behavior of this disordered loop, i.e., the preferred conformations (or conformational ensemble) and interaction potency of this acidic region, seems to be significantly changed due to polyphosphorylation. On the contrary, we should remember a possible role of inorganic polyphosphates themselves in protein quality control: among their possible functions, one can mention chaperone-like function of such polymers [6]. From this point of view, further acidification of the disordered loop by addition of polyphosphate chains might significantly influence the binding unfolded/misfolded client proteins as well as their structure. In other words, this region of HSP90, which represents acidic PASK-motif, serves as a polyanion, which is very potent for the interaction with client proteins and can became even more reactive due to polyphosphorylation; this effect should be the most pronounced for longer chains of polyphosphates attached. Noteworthy, HSP90 interacts with many proteins (including client proteins linked with cancer [16]), and its activity level can influence the evolution and hydrophobicity of these proteins [28]. If polyphosphorylation influences HSP activity or affinity to client proteins, the role of such post-translational modification might be very substantial.

Effect of polyphosphorylation at different tested sites was different, that raises a question of site specificity of the modification. The most common structural determinant of polyphosphorylated proteins is the presence of highly acidic PASK-motif [9], but as we showed in this in silico study, even exact polyphosphorylation site within the one prolonged PASK-motif can matter. In addition, some proteins with no manifested PASK-motif can be polyphosphorylated [8]. On the contrary, since polyphosphorylation is a non-enzymatic post-translational modification (in contrast to a rare lysine phosphorylation [11]), it might strongly depend on not only protein inherent features, but also on environmental conditions such as inorganic polyphosphate level. From this point of view, highly acidic and disordered protein regions are very reactive and therefore potent for polyphosphorylation; location on the protein surface and a good exposition can facilitate the modification of particular lysine residues.

The importance of the nature of the modification site can be illustrated by a comparison of polyphosphorylation effect on two homological proteins, bacterial HtpG and human endoplasmin. It should be noted that the tested polyphosphorylated form of bacterial HSP90 is completely speculative since there is no data on polyphosphorylation of this protein in vivo, although it does not look impossible because of high level of polyphosphates in bacteria and demonstrated cases of polyphosphorylation of proteins with a subtle PASK-domains. However, effect of this modification of HtpG from *E. coli*, which contains only a short beta-hairpin with few acidic residues and few lysine residues instead of the prolonged disordered PASK-motif in eukaryotic HSP90, was less pronounced compared to the effect of polyphosphorylation of human HSP90. Only a direct interaction of polyphosphate chain with adjacent protein regions followed by a slight rotation of the beta-hairpin was detected. On the contrary, polyphosphorylation of the prolonged disordered charged linker in human HSP90 induced significant changes in conformational ensemble that should influence protein behavior because of importance of the modified region.

One of the suggested roles of protein polyphosphorylation is participation in signaling. In this context, a central question with no answer is what is a polyphosphorylation, a particular function or a side effect. Since single phosphorylation of even one residue can dramatically change protein interaction with other macromolecules (as is the case for example for MDM2 E3 ubiquitin ligase [29]), polyphosphorylation, as a thicker (i.e., a large charged chain is attached) modification, is expected to exhibit a greater effect. This hypothesis can be supported by the fact that polyphosphate-accumulating cells demonstrated changes in cellular localization of some proteins [30]; polyphosphorylation of some proteins regulates their functions and involves key cellular pathways (such as yeast interacting pair Nsr1/Top1, nuclear signal recognition 1/topoisomerase 1, which were the first discovered polyphosphorylated proteins [7]). Focusing on HSP90, the effect of polyphosphorylation can be very important, and one can hypothesize that in some conditions associated with a high level of inorganic polyphosphates in cells, polyphosphorylation of HSP90 regulates its interaction with client proteins and, as a result, its activity. This pathway might represent an important response of cell on stresses associated with protein aggregation. Amyloidosis can be a good example of such conditions; the data supporting this idea are discussed in the next paragraph. On the other hand, a non-enzymatic nature of this post-translational modification suggests that it might be just a side effect of a high level of inorganic polyphosphates in cells.

Finally, malfunction of HSP90 chaperones can be associated with progression of neurodegenerative diseases [18,31,32]. Therefore, if polyphosphorylation changes HSP90 behavior and modulates its interaction with amyloidogenic (unfolded) proteins, further study of this effect is relevant for understanding the ways of development of neurodegenerative diseases such as Alzheimer’s disease or Parkinson’s disease and the role of other macromolecules (such as non-protein polyelectrolytes) in these processes. Indeed, although there is no data about any direct links between polyphosphorylation of HSP90 or other proteins and neurodegenerative diseases, inorganic polyphosphates seem to be linked with such diseases. First, there are data about effect of polyphosphates on amyloid aggregation [33,34]. On the other hand, endogenous level of polyphosphates might be altered in case of Alzheimer’s disease or Parkinson’s disease [35] as well as in yeast model of polyglutamine disease [36]. Therefore, it is tempting to hypothesize that inorganic polyphosphates might influence amyloidogenic processes not only directly due to interacting with amyloidogenic proteins, but also indirectly due to polyphosphorylation of the HSP90, which is linked with neurodegenerative diseases and is known to be polyphosphorylated [7]. In other words, the role of HSP90 in development of neurodegenerative diseases can be affected by polyphosphorylation if inorganic polyphosphates participate somehow in amyloidogenic processes.

## Data Availability

Data available on request.
